# Identification of lncRNAs by RNA Sequencing Analysis During *in Vivo* Pre-Implantation Developmental Transformation in the Goat

**DOI:** 10.3389/fgene.2019.01040

**Published:** 2019-10-25

**Authors:** Ying-hui Ling, Qi Zheng, Yun-sheng Li, Meng-hua Sui, Hao Wu, Yun-hai Zhang, Ming-xing Chu, Yue-hui Ma, Fu-gui Fang, Li-na Xu

**Affiliations:** ^1^College of Animal Science and Technology, Anhui Agricultural University, Hefei, China; ^2^Local Animal Genetic Resources Conservation and Biobreeding Laboratory of Anhui Province, Hefei, China; ^3^Key Laboratory of Farm Animal Genetic Resources and Germplasm Innovation of Ministry of Agriculture, Chinese Academy of Agricultural Sciences, Beijing, China; ^4^Institute of Plant Protection and Agro-Products Safety, Anhui Academy of Agricultural Sciences, Hefei, China

**Keywords:** RNA-seq, Goat, long noncoding RNAs, pre-implantation development, zygotic gene activation

## Abstract

Pre-implantation development is a dynamic, complex and precisely regulated process that is critical for mammalian development. There is currently no description of the role of the long noncoding RNAs (lncRNAs) during the pre-implantation stages in the goat. The *in vivo* transcriptomes of oocytes (n = 3) and pre-implantation stages (n=19) at seven developmental stages in the goat were analyzed by RNA sequencing (RNA-Seq). The major zygotic gene activation (ZGA) event was found to occur between the 8- and 16-cell stages in the pre-implantation stages. We identified 5,160 differentially expressed lncRNAs (DELs) in developmental stage comparisons and functional analyses of the major and minor ZGAs. Fourteen lncRNA modules were found corresponding to specific pre-implantation developmental stages by weighted gene co-expression network analysis (WGCNA). A comprehensive analysis of the lncRNAs at each developmental transition of high correlation modules was done. We also identified lncRNA-mRNA networks and hub-lncRNAs for the high correlation modules at each stage. The extensive association of lncRNA target genes with other embryonic genes suggests an important regulatory role for lncRNAs in embryonic development. These data will facilitate further exploration of the role of lncRNAs in the developmental transformation in the pre-implantation stage.

## Introduction

Pre-implantation development comprises complex and dynamic regulatory processes involving specific and stable gene expression patterns that maintain the viability of the embryo. During different embryonic stages, highly complex tissues are composed of different cell types that are formed by cell fate and cell differentiation (Lokken and Ralston, 2016; Bissiere et al., 2018). Analysis of the spatiotemporal patterns of gene expression in goat pre-implantation stages is therefore essential for clarifying early developmental processes in this species. The key stage in the transition from germ cells to embryonic development is zygotic gene activation (ZGA), which induces developmental blocks of embryonic development (Lee et al., 2014). The timing of mammalian ZGA process is species-specific; it occurs from the point of oocyte maturation until mRNA transcriptional activity in the embryo. The initiation of major ZGA events have been reported to be the 2-cell stage in mouse (Xue et al., 2013), 4-cell stage in pig (Cao et al., 2014), and 4-cell to 8-cell stage in human (Xue et al., 2013; Yan et al., 2013). Previous studies had reported that ZGA-related genes begin to be expressed in the 8-cell to 16-cell stage of goats (Ma et al., 2014; Deng et al., 2018). Recent study of the developmental block of cultured of goat *in vitro* suggested that cell development stops at the 8-cell stage, and further verification by RNA-seq has indicated that it occurs between the 4- and 8-cell stage (Deng et al., 2018).

Encoded protein sequences represent less than 2% of the mammalian genome whereas a much larger fraction of this genome is transcribed into what is known as noncoding RNAs (ncRNAs) (Agliano et al., 2019). Many ncRNAs are expressed in pre-implantation stages and play an important role in fertilization and appropriate embryonic development (Hamazaki et al., 2015; Yuan et al., 2016; Vallot et al., 2017). Long ncRNAs (lncRNAs) are among the largest ncRNAs in vertebrates and are broadly defined as noncoding transcripts of greater than 200 nucleotides (Sun and Kraus, 2013; Agliano et al., 2019). For example, Trincr1 binds to TRIM71 to inhibit FGF/ERK signaling in embryonic stem cells to coordinate cell fate specifications (Li et al., 2019). Most of the studies on the expression of lncRNAs in pre-implantation stages have been focused on humans (Kurian et al., 2015), mice (Karlic et al., 2017), and pigs (Zhong et al., 2018). In comparable functional studies of oocyte and pre-implantation cells lncRNAs in the goat are limited.

The domestic goat (*Capra hircus*) is one of the most important commercially farmed animals that produces a variety of products, including meat, milk, and skins (Guan et al., 2016). Moreover, various established reproductive biotechnologies have made the goat a significant species used in agriculture and transgenic breeding research (Baguisi et al., 1999; Bao et al., 2016). The emergence of low input high throughput sequencing technologies has enabled the transcriptome to be determined from oocytes and pre-implantation cells at different stages of development in the goat.

In our current study, the transcriptomes of seven pre-implantation developmental stages of goat, including *in vivo* metaphase II mature oocytes and the 2-cell, 4-cell, 8-cell, 16-cell, morula and blastocyst stages, were sequenced using low input high throughput RNA-seq. This analysis identified the timing of goat ZGA and identified the differential expression of lncRNAs in oocytes and pre-implantation stages, and thereby revealed the role of the lncRNAs in ZGA event. Further, we constructed a WGCNA network to identify the lncRNAs and lncRNA-mRNA networks that are highly correlated at each stage, and to identify the hub-lncRNAs in all pre-implantation stages. This compilation-specific network analysis has given us a more comprehensive understanding of the functional transition of lncRNAs at specific stages of pre-implantation in the goat.

## Materials and Methods

### Goat Pre-Implantation Stages Material

Female Anhui white goats (AWGs) were farm-raised by the Boda Company (Baogong Town, Feidong County, Hefei, China) under a unified field management system. All experimental animals were estrus-synchronized by treatment with EAZI-Breed CIDR (CIDR, Hamilton, New Zealand) for 12 days and superovulated prior to CIDR removal. The estrus test was performed 12h after stopping CIDR, and artificial insemination was performed on the female AWGs that were estrus at the same time. After 36–48, 56–60, 87–92, 97–100, and 109–112 h of mating, oocytes and 2-cell, 4-cell, 8-cell, and 16-cell cells were flushed from the oviduct. Morulae and blastocysts were obtained from the uterus after 152–156 and 212–218 h, respectively. A total of 21 samples were obtained in these seven stages, and each stage of the sample had three replicates. Oocytes and pre-implantation cells were washed several times in 1% DBPS solution. Five obtained oocytes and pre-implantation cells at each stage were pooled and snap frozen in liquid nitrogen.

### RNA Isolation, Library Preparation, and Sequencing

RNA isolation, library construction and sequencing were done by Novogene Co. Ltd. (Beijing, China). Total RNA from individual oocytes and pre-implantation cells was isolated using TRIzol reagent (Invitrogen, Carlsbad, CA); and RNA was co-precipitated with linear acrylamide (Ambion, Texas, USA). RNA integrity was evaluated on 1% agarose gel. RNA purity was checked using a NanoPhotometer (Implen, CA, USA). RNA concentrations were measured using a Qubit® RNA Assay Kit and Qubit® 2.0 Flurometer (Life Technologies, CA, USA). We then used 3 ng of RNA as the base material for cDNA sample preparation, and purified cDNA was obtained and detected on an Agilent Bioanalyzer 2100 system (Agilent technologies, CA, USA). The clustering of the index-coded samples was performed on a cBot Cluster Generation System using TruSeq PE Cluster Kit v3-cBot-HS (Illumia, CA, USA) in accordance with the manufacturer’s instructions. After cluster generation, the libraries were sequenced on an Illumina Hiseq 2500 platform and 150 bp paired-end reads were generated (**Table S1**).

### Data Analysis

Raw data (raw reads) in a fastq format were first processed through in-house perl scripts (ng-qc). All the linker sequences in the raw data would be removed, ng-qc parameter: -L 20 -p 0.5 (-L, lowest quality value, -p parameter of low-quality reads.-L20 –p 0.5 was the low-quality base ratio allowed by the specified reads; the default was 0.5. This means that the number of bases of quality value ≤ -L parameter (Baguisi et al., 1999)/reads length ≥ 0.5 represented low quality reads). In addition, entering the adapter sequence in the ng-qc software would be removed by sequence matching. Clean data (clean reads) were obtained by removing reads from the raw data that contained adapters, reads with undetermined base content greater than 10%, and low-quality reads (**Table S1**). Moreover, clean reads satisfied the conditions of Q20 > 90% and Q30 > 85%. This meant that reads with a base error rate of less than 0.01 account for more than 90% of all reads, and reads with an error rate of less than 0.001 account for more than 85% of all reads. The *Capra hircus* reference genome and gene model annotation files for this study can be accessed at (The Capra hircus reference gene model annotation file; The Capra hircus reference genome model annotation file). An index of the reference genome was built using Bowtie v2.0.6 (Langmead and Salzberg, 2012) and paired-end clean reads were aligned to this using TopHat v2.0.9, both with default parameters (Trapnell et al., 2009). The mapped reads of each sample were assembled using both Scripture (beta2) (Trapnell et al., 2010) and Cufflinks (v2.1.1) (Guttman et al., 2010) *via* a reference-based approach. Scripture used a statistical segmentation model to distinguish expressed loci from experimental noise and spliced reads to assemble expressed segments. It reported all statistically expressed isoforms in a given locus. Cufflinks uses a probabilistic model to simultaneously assemble and quantify the expression level of a minimal set of isoforms that provides a maximum likelihood explanation of the expression data in a given locus. Scripture was run with default parameters, Cufflinks was run with ‘min-frags-per-transfrag = 0’ and ‘–library-type’; other parameters were set as default.

Based on the splicing results, the structural characteristics of lncRNA and the functional characteristics of non-encoded proteins, a 5-step screening was performed, and the lncRNAs obtained were used as the final candidate lncRNA set for subsequent analysis. First, the transcripts spliced from all samples were combined using cuffcompare to screen for transcripts of unknown molecular orientation. Second, we chose transcripts with transcript length ≥ 200 bp and exon number ≥ 2. Then, we calculated the read coverage of each transcript by cufflinks and selected a transcript with a coverage of ≥ 3 reads in at least one sample. Next, the transcript obtained in the previous step was first compared with the known lncRNA by cuffcompare to obtain the same transcript as the known lncRNA. This part of the transcript was directly included in the final lncRNA set and no further screening was performed. Finally, the transcripts of the candidate lincRNA, intronic lncRNA, and anti-sense lncRNA type were screened by comparison with known mRNAs and using the class_code information in the cuffcompare analysis results (**Table S2**) (Cuffcompare program of Cufflinks).

Then, transcripts with coding potential were filtered by Coding-Non-Coding-Index (CNCI) (v2) (Sun et al., 2013), Coding Potential Calculator (CPC) (0.9-r2) (Kong et al., 2007), and Pfam-scan (PFAM) (v1.3) (31), and the noncoding transcripts were selected as our candidate lncRNAs. The CNCI parameters include –f input transcriptome sequence file, –o data output path, –p 1 (number of cpu) and -m ve (specified mode, ve is vertebrate). The index in the CNCI prediction result would be labeled as coding or noncoding (Sun et al., 2013). CPC (0.9-r2), used with default parameters, searched sequences with known protein sequence databases to elucidate both coding and non-coding transcripts (Kong et al., 2007). In addition, we translated each transcript in all three possible frames and used PFAM (v1.3) to identify the presence of any known protein family domain recorded in the Pfam database (release 27; used Pfam B). A transcript with a PFAM hit will be excluded in the following steps. Pfam searches use default parameters of -E 0.001 -domE 0.001 and -cpu 2 (CPU set to 2) (Bateman et al., 2004; Punta et al., 2012).

### Quantification of Gene Expression and Differential Expression Analysis

Cuffdiff (v2.1.1) was used to calculate the FPKM (fragments per kilo-base of exon per million fragments mapped) of both the lncRNAs and coding genes in each sample (Trapnell et al., 2010). Gene FPKMs were computed by summing those for the transcripts in each gene. Principal Component Analysis (PCA) was conducted using R and heat map/cluster analysis using the Morpheus free online platform (). The applied statistical procedures used a negative binomial distribution model in Cuffdiff to determine differentially expressed transcripts (Trapnell et al., 2010). For biological replicates, transcripts or genes with a *P*-adj < 0.05 were assigned as differentially expressed.

### Target Gene Prediction and Functional Analysis

The interaction of lncRNA with a nearby target gene was called *cis*- action. We searched for coding genes 10 kb upstream and downstream of each lncRNA. Candidate target genes for *trans*-acting lncRNAs were predicted based on co-expression. The Pearson correlation coefficient method was used to analyze correlations between mRNAs and lncRNAs. mRNAs with absolute correlation value greater than 0.95 were considered to be target genes for lncRNAs. LncRNA-mRNA networks were constructed using Cytospace (Cytoscpace software). Gene Ontology (GO) is a classification system for internationally standardized gene functions that provides a controlled vocabulary to comprehensively describe the properties of genes and their products. GO enrichment analysis of differentially expressed genes or lncRNA target genes was performed using the GO-seq R package, in which gene length bias was corrected (Young et al., 2010). GO terms with *P-*value < 0.05 were considered to indicate significant enrichment of those respective differential genes. Bubble charts were constructed using the OmicShare platform for data analysis (Omicshare tools).

### Weighted Gene Co-Expression Network Analysis (WGCNA)

Differentially expressed lncRNAs with an FPKM > 0.01 between all pre-implantation cells development stages were selected, and the lncRNA co-expression network was then constructed using R package WGCNA (Langfelder and Horvath, 2008). A signed weighted correlation network was generated by first creating a matrix of Pearson correlation coefficients between all pairs of genes across the measured samples. An adjacency matrix was then transformed into a topological overlap matrix (TOM) to minimize the effects of noise and spurious associations. To define modules as branches, we employed the Dynamic Tree Cut algorithm with default parameters to cut the hierarchal clustering tree (Langfelder et al., 2008).

### Quantitative RT-PCR

QRT-PCR was performed using GoTaq qPCR Master Mix (Promega, Madison, WI) and Real-time Thermal Cycler 5100 (Thermo, Shanghai, China). The primer pairs used in the PCR amplifications were synthesized by the Beijing Genomics Institute and are listed in **Table S3**. The GAPDH housekeeping gene was amplified as a control (Li et al., 2019). The target sequence levels were normalized to the reference sequence and calculated as 2^−ΔΔCt^. Statistical analysis of the normalized data was then conducted using SPSS version 19.0 for Windows (SPSS Statistics). Data are presented as means ± SEM. Data were considered statistically significant at *P-*value < 0.05.

## Results

### Transcriptome Reconstruction From RNA-Seq Data

**Figure 1:**
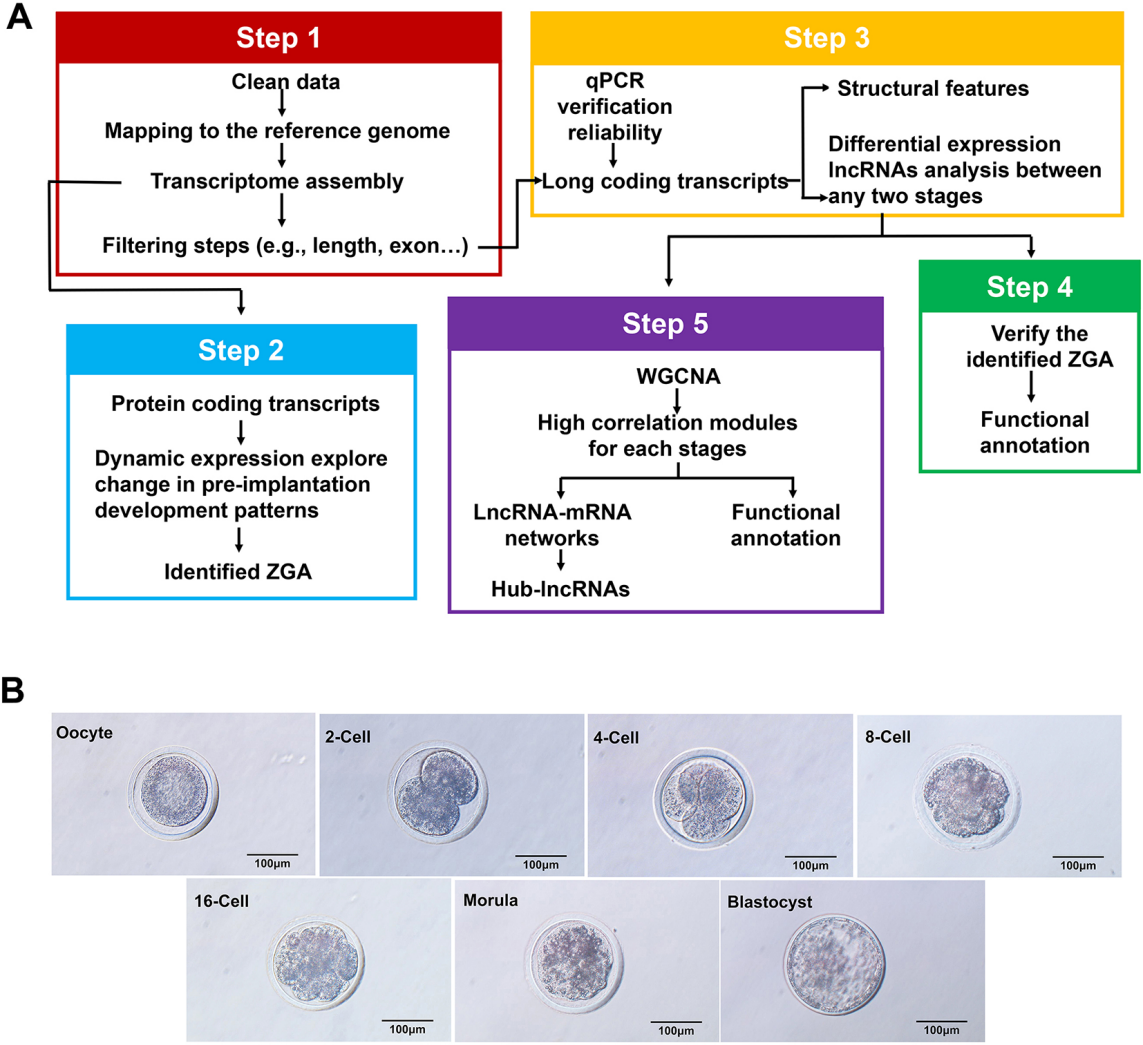
Comprehensive catalog of the lncRNAs in goat oocytes and embryos at different stages. **(A)** Schematic of the experimental design method for the identification of expressed protein-coding transcripts and IncRNAs. Scale bar, 100 µm. **(B)** Microscopy imaging of mature goat oocytes and embryos at the metaphase II oocyte, 2-cell, 4-cell, 8-cell, 16-cell, morula and late blastocyst stages.

We collected 21 samples from Anhui white goats after superovulation treatment and then performed RNA-seq analysis (**Figure 1A**). The cells were obtained from seven crucial stages i.e. metaphase II oocytes and 2-cell, 4-cell, 8-cell, 16-cell, morula and blastocyst stage (**Figure 1B**). An Illumina HiSeq 2500 sequencer was used and 290.8 GB of clean sequencing data were generated from the 21 samples, with an average of 92.1 million total mapped reads per stage (**Table S1**).

### Dynamic Patterns of Protein-Coding Transcript Profiles

**Figure 2:**
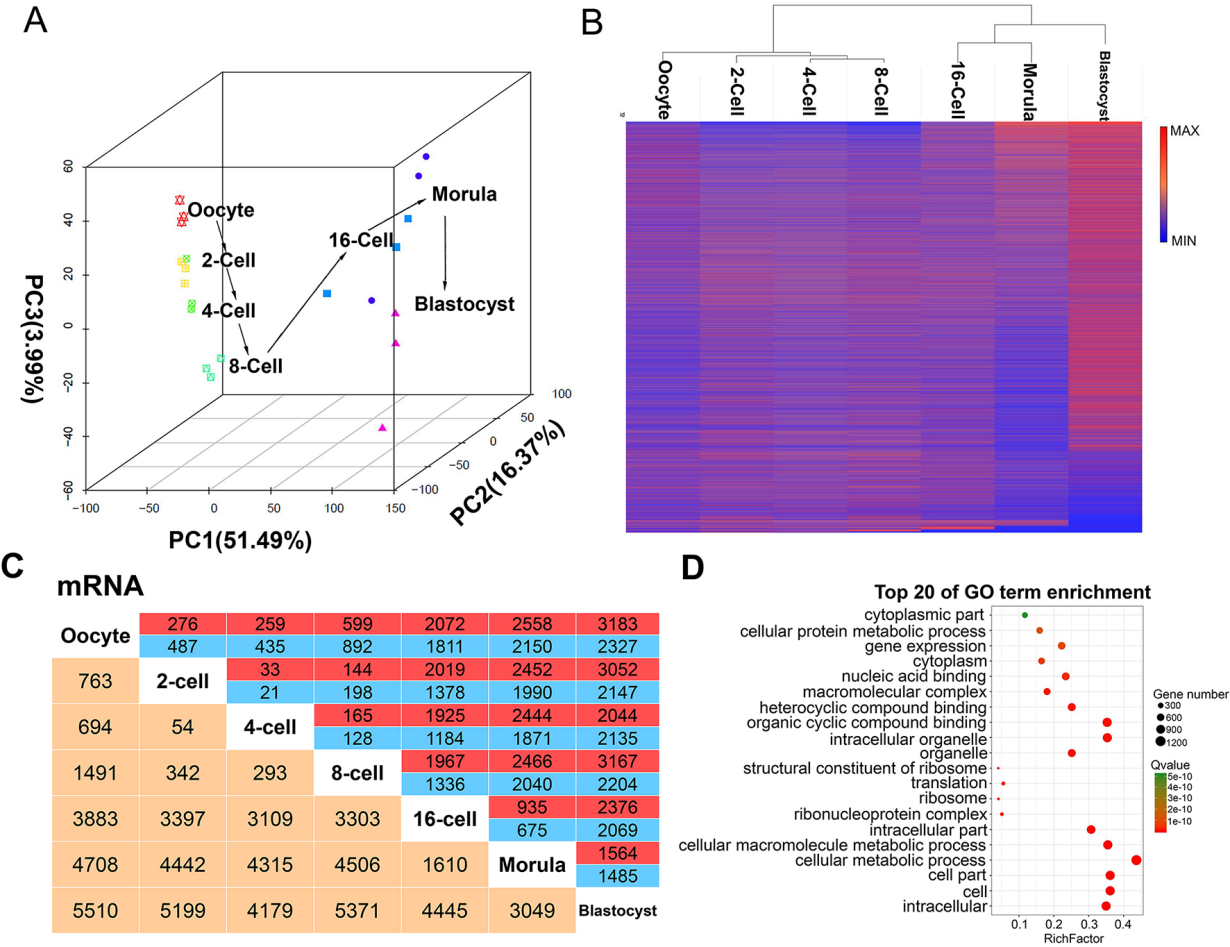
mRNA expression patterns during pre-implantation development in the goat. **(A)** Principal component analysis (PCA) of mRNAs in 21 goat pre-implantation development samples at 7 different stages. The same color represents the same stage. The arrows indicate the direction of development between successive muscle stages. **(B)** Hierarchical clustering heat map of mRNAs by sample. Red, relatively high expression; blue, relatively low expression. **(C)** Number of differentially expressed mRNA showing up- (red) or down- (blue) regulation during development. Yellow, total number of differentially expressed mRNAs between any two stages. **(D)** Top 20 enriched GO terms for the differentially expressed mRNA between the 8- and 16-cell stage.

A total of 29,608 protein-coding transcripts were identified during the seven goat pre-implantation stages (**Table S4**). Principal component analysis was used to capture the expression of transcripts from the oocyte to blastocyst development stages. Oocytes and pre-implantation cells at the same stage were found to cluster with each other, except that one 4-cell stage was clustered in the 2-cell stage, and one morula was clustered in the 16-cell stage (**Figure 2A**). The greatest changes in gene expression were observed in the 8- and 16-cell stages, possibly due to maternal-zygote transitions during this period. Hierarchical clustering also yielded similar intra- and inter-phase expression patterns (**Figure 2B**). All of the stages of goat development were divided into two processes: from the oocyte to 8-cell stage and from the 16-cell to blastocyst stage. Two other minor ZGAs were found to occur between the oocyte and the 2-, 4-, and 8-cell stages, and between the morula and the blastocyst stage (**Figure 2A, B**). Moreover, 10,197 differentially expressed mRNAs were identified, and the largest change was also observed between 8-cell and 16-cell in two consecutive comparison groups (**Figure 2C**). Functional analysis of these differentially expressed mRNAs was enriched in 110 GO terms, including “metabolic,” “binding,” and “biosynthetic processes,” as well as “enzymatic activity,” such as “cell part,” “cellular macromolecule metabolic process,” “cellular biosynthetic process,” “ribonucleotide binding,” “phosphoprotein phosphatase activity” and other terms. This stratification indicated that goat ZGA occurs between the 8- and 16-cell stages (**Figure 2D**).

### Genomic Structural Features of Goat lncRNAs

**Figure 3:**
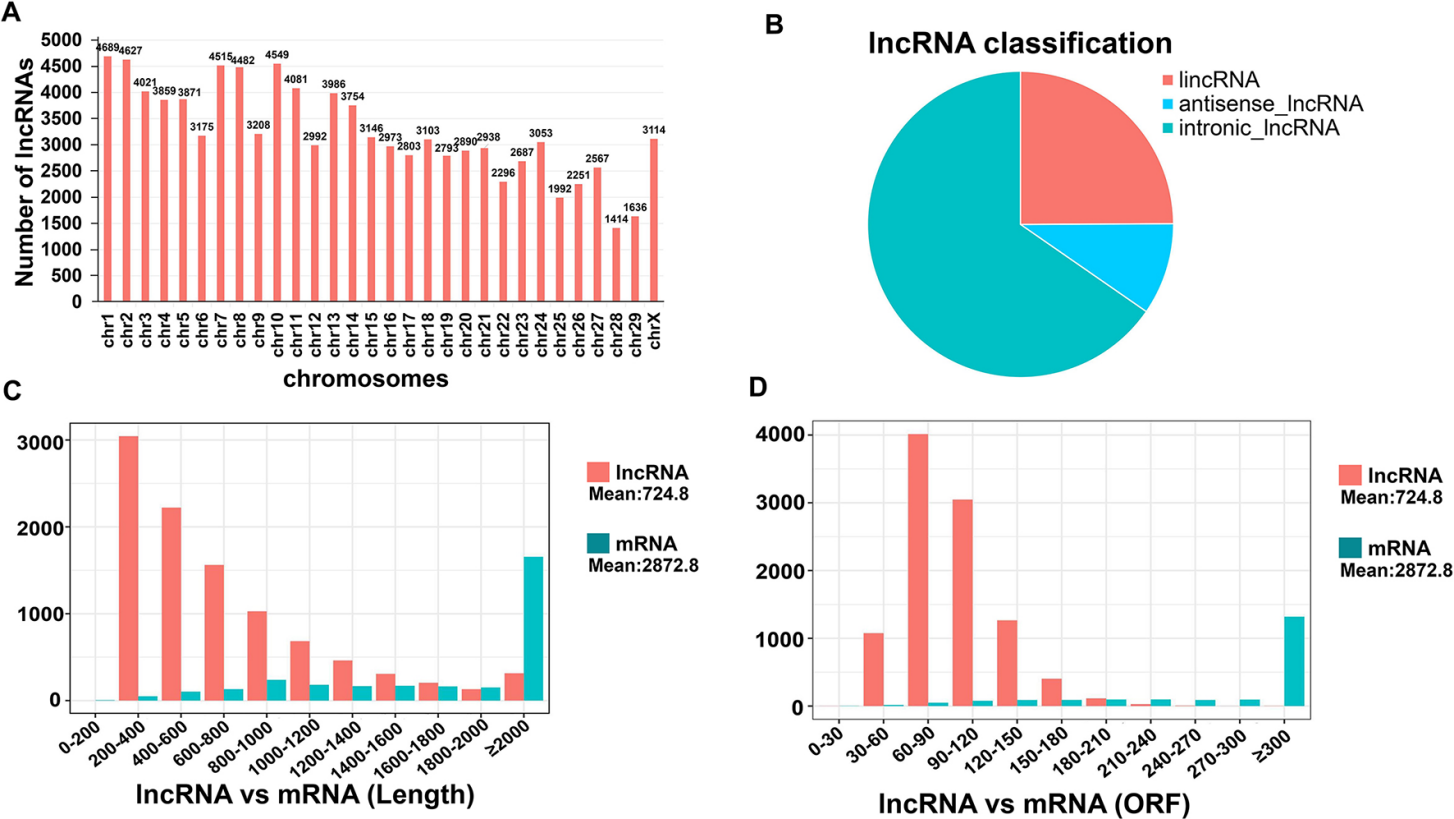
Chromosome distribution and characteristics of lncRNAs in goat pre-implantation development. **(A)** Distribution of all identified lncRNAs in goat chromosomes. **(B)** Classification of lncRNAs. **(C**–**D)** Transcript length and open reading frame (ORF) length distribution of transcripts for all lncRNAs and mRNAs in goat skeletal muscle. Orange, lncRNA; blue, mRNA.

CNCI, CPC, and PFAM were used to remove potential encoded transcripts after a highly stringent filtering pipeline was applied (**Figure S1A**). A final total of 99,621 putative lncRNAs were retained (**Figure S1B**). Most of these lncRNAs (97.8%) were found to be distributed on all chromosomes except for the Y chromosome (**Table S5**). We further found that goat chromosomes 1, 2, 7 and 10 produce more lncRNAs (> 4500) than any of the others (**Figure 3A**). The identified lncRNAs were mainly divided into three categories: lincRNA, antisense lncRNA, and intronic lncRNA. Among them, intronic lncRNA was the most abundant, accounting for 65.3%, followed by lincRNA (24.9%) (**Figure 3B**). We speculated from this that these 4 chromosomes make the major contribution to the role of the lncRNAs in oocytes and pre-implantation cells growth. Combining multiple structural features to maximize our understanding of lncRNA and mRNA functions is important. The lncRNAs have an average length of 724.75 bp, which is shorter than the average protein-coding transcript length of 2872.80 bp in goat (**Figure 3C**). In addition, the lncRNAs in our current dataset were shorter than the protein-coding genes in terms of the ORF length (mean 93.78 bp vs. 520.22 bp, respectively) (**Figure 3D**).

### Dynamic Expression of Differentially Expressed lncRNAs

**Figure 4:**
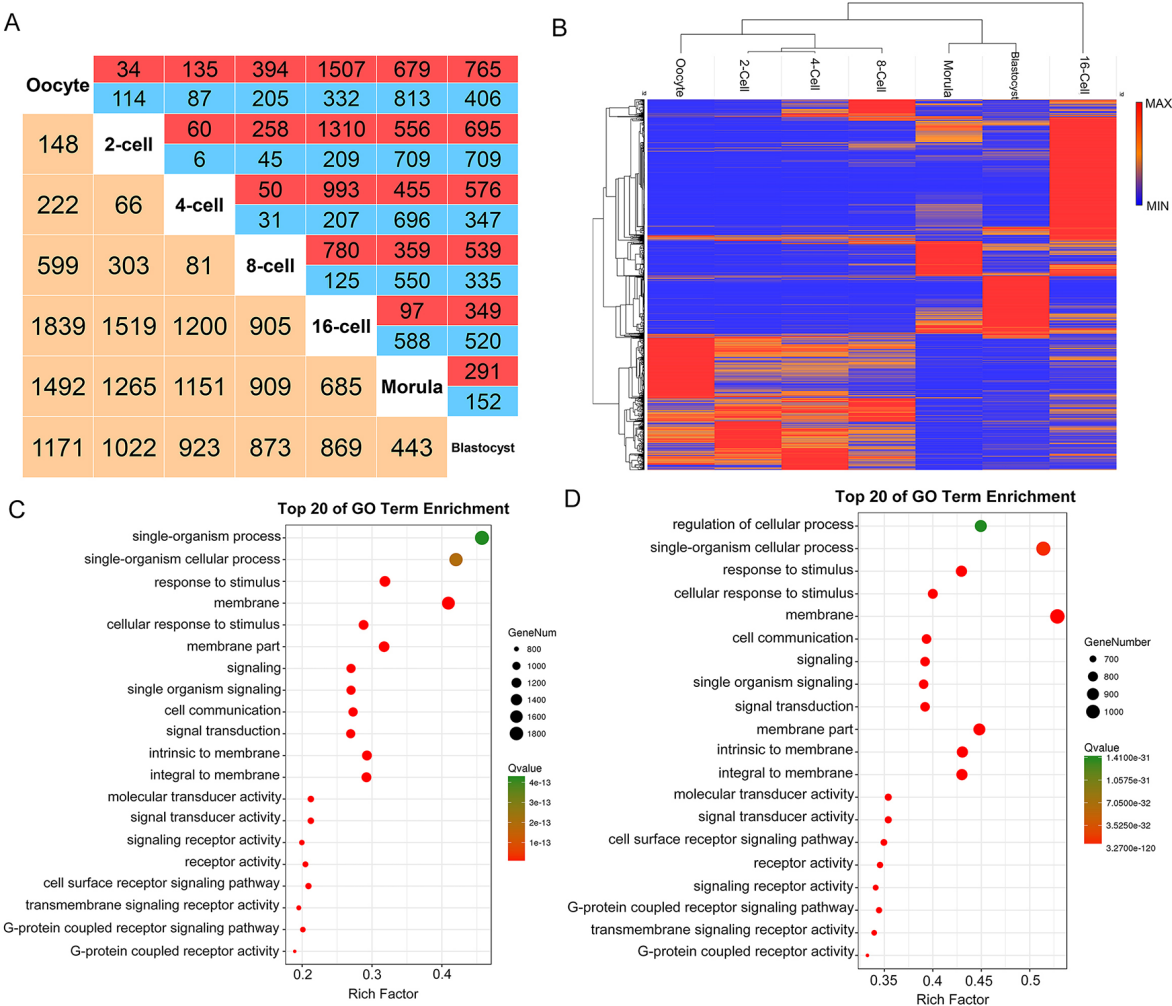
LncRNA expression patterns during pre-implantation development in the goat. **(A)** Number of differentially expressed lncRNAs (DELs) showing up- (red) or down- (blue) regulation during development. Yellow, total number of DELs between any two stages. **(B)** Hierarchical clustering heat map of DELs by samples. Red, relatively high expression; blue, relatively low expression. **(C**–**D)** Top 20 enriched GO terms for the DELs between the 8- and 16-cell stage, and between the oocyte and 2-cell stage, respectively. Green, relatively high expression; red, relatively low expression.

We examined the differential expression of lncRNAs (*P-*adj < 0.05) between all stages of goat pre-implantation development. We identified 5160 differentially expressed lncRNAs (DELs) in these seven stages (**Figure 4A**, **Table S6**). In an unbiased hieratical clustering of these DELs, the 16-cell stage produced the largest differences from the other stages, which also confirmed the time of the major ZGA occurrence (Figures 4A, B). Interestingly, the gene expression profiles between the 2-, 4-, and 8-cell in the goat were similar. Differing from the protein-encoding transcripts, the clustering of DELs revealed that oocytes were separated from the first two cleavage events (2, 4, 8-cell stages) and the 16-cell stage was separated from the morula (**Figure 4B**). Hence, we mainly focused on the DELs between the 8- and 16-cell stages and between the oocytes and 2-cell stage.

In the major ZGA event in the goat pre-implantation stage, 905 DELs were found to be generated between the 8- and 16- cell stages, of which 780 were up-regulated and 125 were down-regulated. These DELs were enriched (*P-*adj < 0.05) in 24 GO terms, such as “G-protein coupled receptor activity,” “G-protein coupled receptor signaling pathway,” “transmembrane signaling receptor activity,” and others (**Figure 4C**, **Table S7**). The minor ZGA from the oocyte to the 2-cell stage produced 148 DELs, 34 of which were up-regulated and 114 were down-regulated. Functional analysis of these two transformation stages included the “G-protein coupled receptor signaling pathway,” “signaling receptor activity,” “cell surface receptor signaling pathway,” and others (**Figure 4D**, **Table S8**). Overall, these RNA-seq data provided an *in vivo* overview of the role of lncRNAs in ZGA waves in the goat pre-implantation stages.

### WGCNA Revealing the Role of the DELs in the Developmental Transformation Leading to Pre-Implantation in the Goat

**Figure 5:**
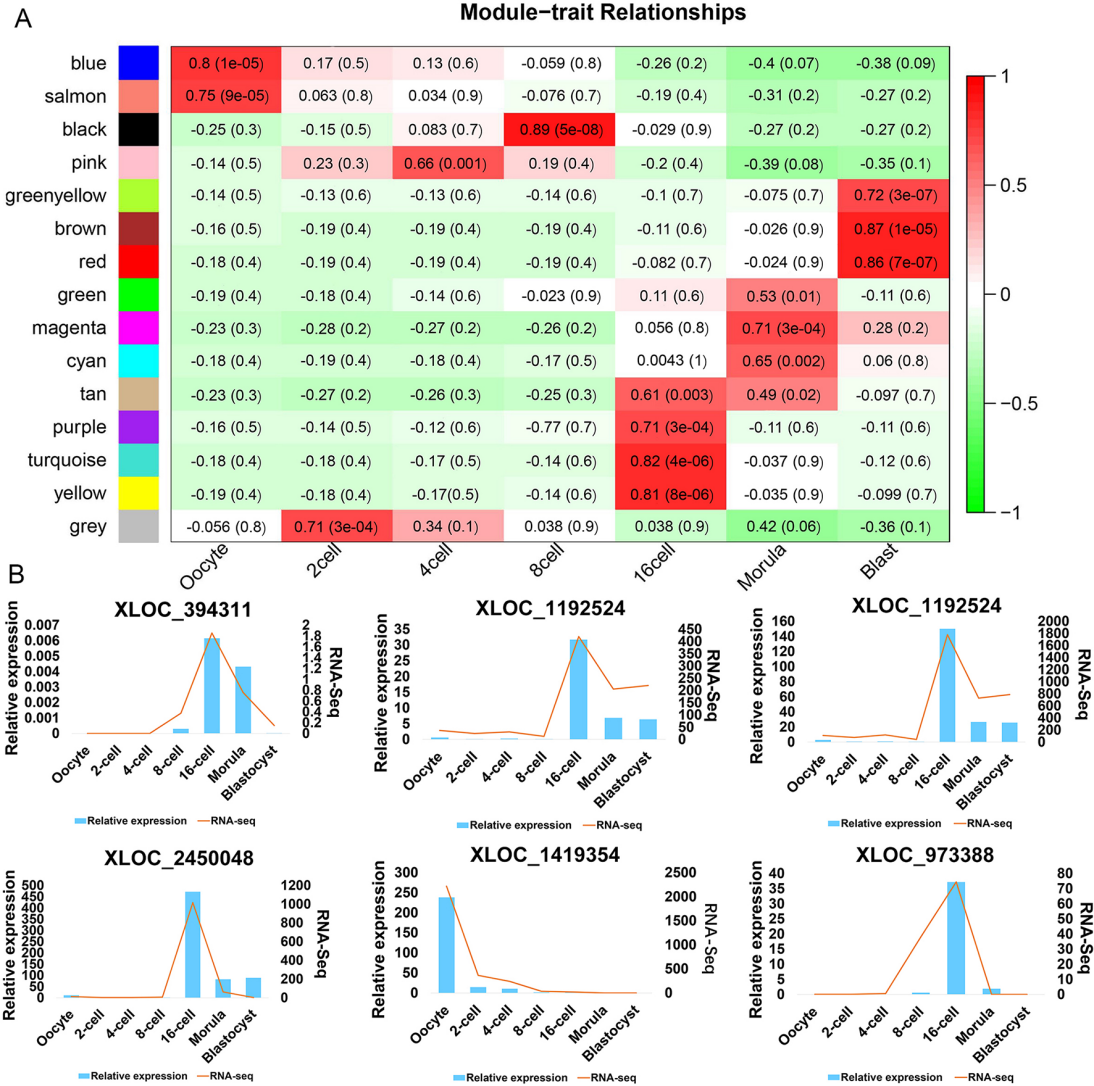
LncRNA expression modules determined by WGCNA **(A)** Hierarchical clustering heat map of DELs (with an FPKM > 0.01 in at least one sample during the seven stages). **(B)** qPCR (bar chart, blue) and RNA-seq expression (line chart, orange) validation of the indicated lncRNAs.

There has been no prior study describing the expression profiling of lncRNAs during goat oocyte and pre-implantation development. In addition, little functional research on these lncRNAs has been reported. To investigate the potential role of DELs in pre-implantation development, WGCNA was performed on 4761 DELs that had been filtered (FPKM > 0.01 during at least one developmental stage) and correlation analysis was conducted on the obtained modules (**Table S9**). This analysis revealed that goat DELs prior to implantation can be divided into 15 modules (denoted in the figure using different colors), 14 of which were highly correlated (correlation > 0.6, *P*-value < 0.05) with a specific developmental stage (**Figure 5A**, **Figure S2**). Interestingly, each preimplantation period had corresponding high expression modules. Moreover, six lncRNAs were randomly identified from stage-specific modules by qRT-PCR analysis (**Figure 5B**).

**Figure 6:**
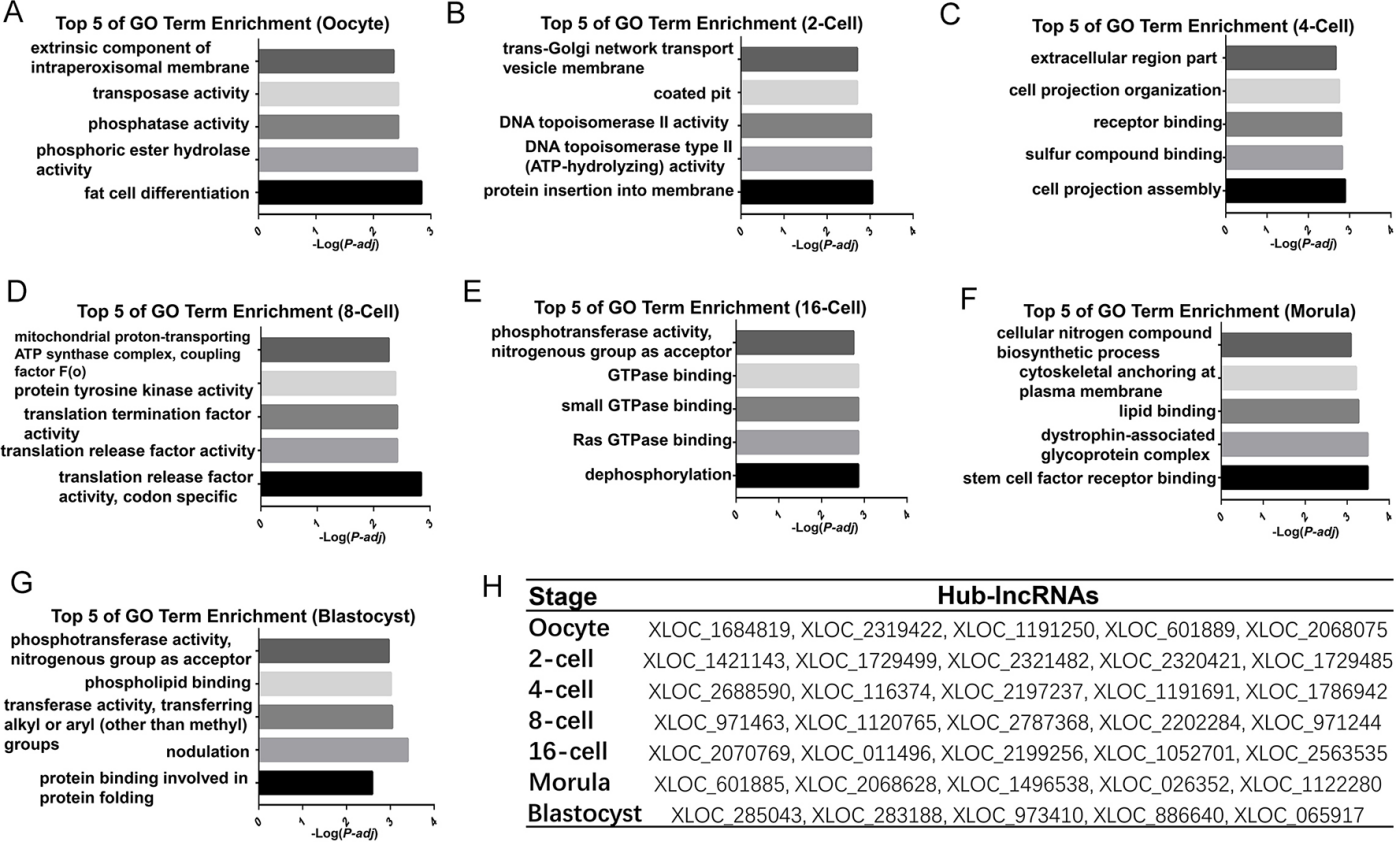
Functional prediction of the high correlation modules involved in pre-implantation development in the goat **(A**–**G)** Bar plots showing the top 5 GO enrichment terms in the high correlation modules of each developmental stage in the goat. The length of the bars indicates the significance (-log_10_ transferred *P*-value). **(H)** Identified top5 degree hub-lncRNAs corresponding to the development stage.

To explore DEL functions in the goat pre-implantation period, GO terminology enrichment analysis was performed for the different aforementioned modules. Interestingly, our analyses of the functions in these modules revealed a sequential progression of stage-specific core genetic networks (**Table S10**). Initially, the functional enrichment of oocyte modules (blue and salmon) included “transposase activity,” “transposition,” “DNA-mediated, fat cell differentiation,” and others (**Figure 6A**). The functional processes migrated from “protein insertion into membrane,” “DNA topoisomerase II activity,” and others at the 2-cell (gray) stage, to “cell projection assembly,” “cellular developmental process,” and others in the 4-cell (pink) stage, and then to “translation release factor activity,” “translation termination factor activity,” and others at the 8-cell (black) stage (**Figures 6B–F**). Functional analysis of the 16-cell stage goat modules (tan, purple, turquoise, and yellow), which occurs after the major ZGA, revealed the enrichment of 317 GO terms, including “dephosphorylation,” “Ras GTPase binding,” “small GTPase binding,” and others (**Figure 6E**). The two other distinct major stages included “phosphoric ester hydrolase activity,” “stem cell factor receptor binding,” and others in the morula stage, and “protein serine/threonine kinase activity,” “protein binding involved in protein folding” and others at the blastocyst stage (**Figure 6G**). Our current data thus provide the first comprehensive lncRNAs analysis of oocytes and pre-implantation stages in the goat.

To further identify lncRNAs that may play important regulatory roles in these core genetic networks, we screened the lncRNAs with the top five of “degree” as hub-lncRNAs based on the lncRNA-mRNA networks (**Figure 6**, **Figure S3**). Interestingly, most of the lncRNAs were aggregated at the 16-cell stage, which occurs after the major ZGA and produces 49919 lncRNA-mRNA pairs. Moreover, target genes for hub-lncRNAs have been identified as important participants in mammalian pre-implantation development (Pasternak et al., 2016; Daldello et al., 2019). For example, *BTG anti-proliferation factor 4* (*BTG4*) was targeted by hub-lncRNAs in goat oocyte high correlation modules, including XLOC_1684819, XLOC_2068075, and XLOC_601889 (**Table S11**). *Cyclin B2* (*CCNB2*) was also targeted by XLOC_1684819 in the oocyte stage (**Table S11**). Moreover, the top 5 hub-lncRNAs in the 6-cell stage goat all target *activating transcription factor 1*(*ATF1*), which has proved to be one of the key regulators of the ZGA (**Table S11**). These results indicate that the hub-lncRNAs we identified in our current WGCNA may have a critical regulatory role in the pre-implantation developmental stage of the goat.

## Discussion

The major ZGA event is the first important step in the successful initiation of mammalian pre-implantation as it results in the formation of implantable cells. This process is highly dynamic and complex, and an appropriate ZGA is essential for the normal development of the embryo (Wong et al., 2010). However, the timing of ZGA occurrences varied from species to species (Cao et al., 2014; Boroviak et al., 2018). Notably, no comprehensive lncRNA datasets have been available previously for goat pre-implantation stages. In our present study, RNA-Seq was used to analyze the transcriptome and lncRNA profiles during goat pre-implantation. The major ZGA in goat development was found in our present experiments to occur in the 8- to 16- cell stages. This was in contrast to the recent findings by Deng et al. (2018), which reported that the 8-cell stage goat stopped developing in *in vitro* developmental block cultures and showed that ZGA occurred in the 4- and 8-cell stages *in vitro*. However, other studies had shown that the timing of ZGA onset in pre-implantation cells was different between *in vitro* and *in vivo* (Misirlioglu et al., 2006; Graf et al., 2014).

We additionally explored the role of the DELs (n = 5,160) in the pre-implantation process in the goat. The functions of these molecules were identified in major and minor ZGA events that occur in the 8- to 16-cell stage and from the oocyte to 2-cell stage, respectively. The lncRNAs involved in both ZGAs were found to be enriched in “G-protein coupled receptor activity,” “G-protein coupled receptor signaling pathway,” and other functions related to membrane transduction and biological regulation. It is well known that G-protein coupled receptors play a key role in cell self-renewal, differentiation, and signal transduction (Kobayashi et al., 2010; Rutz and Klein, 2015). Our current findings thus revealed that the lncRNAs regulate the cell membrane and its receptors during the ZGA to transduce extracellular physical and chemical signals, and thus play a role in the physiological activities of this process.

We further explored high-correlation lncRNAs at each goat stage and identified each stage of hub-lncRNA according to the lncRNA-mRNA network. The functions of the lncRNAs in these modules migrated from “transposase activity” in oocytes, to “protein insertion into membrane” during the 2-cell stage, to “cell projection assembly” at the 4- cell stage and “translation release factor activity” at the 8-cell stage, to”dephosphorylation” at the 16-cell stage, to “phosphoric ester hydrolase activity” in the morulae, and finally to “protein serine/threonine kinase activity” in the blastocyst. The transformation of the target gene enrichment function at each stage reveals the previously little-known developmental planning role of lncRNAs in goat pre-implantation cells. Furthermore, based on the lncRNA-mRNA networks in the modules and their high correlation with specific development stages, we screened for hub-lncRNAs that are potential key regulators of each pre-implantation stage during goat pre-implantation development. For example, *BTG4*, targeted by XLOC_1684819, XLOC_2068075, and XLOC_601889 lncRNAs, is a meiotic cell cycle-coupled maternal-zygotic-transition licensing factor in oocytes (Pasternak et al., 2016). *BTG4-null* female mice produce morphologically normal oocytes but are infertile due to early developmental arrest (Yu et al., 2016). *CCNB2*, targeted by XLOC_1684819, was also required for progression through meiosis in the oocyte stage (Daldello et al., 2019). Additionally, top 5 hub-lncRNAs in the 16-cell stage goat all target *ATF1*, which might prove to be one of the key regulators of the major ZGA. The presence of activated *ATF1* within the mouse nucleus at the time of ZGA indicates that this transcription factor is a priority target and a key regulator of this event (Jin and O'Neill, 2014; Orozco-Lucero et al., 2017). The DELs that highly correlate with each stage of pre-implantation transformation provides a guide for future studies of the lncRNAs that function in goat pre-implantation development. In addition, the identification of hub-lncRNAs in *in vivo* pre-implantation cells provides a valuable resource for further study of the molecular mechanisms underlying pre-implantation development.

## Conclusion

The *in vivo* transcriptome of metaphase II oocytes, 2-, 4-, 8-, and 16-cell stage cells, and the morula and blastocyst in the goat were analyzed by RNA-Seq. The expression profile of the protein-coding genes indicates that the main ZGA occurs between the 8- and 16-cell stages. The expression profile of the DELs was also verified and these molecules play an important role in the transport and transduction of various substances during the ZGA. In addition, we described the functional continuity of the core genetic network specific for goat pre-implantation developmental stages and identify five hub-lncRNAs in each stage. The role of lncRNA in goat oocytes and pre-implantation development had not been fully elucidated, and our current findings provided valuable resources for future research.

## Data Availability Statement

The datasets analyzed for this study can be found in the SRA database. The Accession number is PRJNA543590.

## Ethics Statement

This study was carried out in accordance with the principles of the Basel Declaration and recommendations of the Guide for the Care and Use of Laboratory Animals (http://grants1.nih.gov/grants/olaw/references/phspol.htm). The protocol was approved by the ethics committee of Anhui Agricultural University under permit No. AHAU20101025.

## Author Contributions

Y-hL and Y-sL conceived the study, and developed hypothesis and research question. QZ, M-hS, and HW participated in the collection and processing of materials. QZ and Y-hL analyzed and interpreted the patient data. QZ carried out qRT-PCR and analyzed data. QZ, Y-hL, and Y-sL participated in the drafting and revision of the manuscript. Y-hZ, HW, Y-hL, M-xC, Y-hM, F-gF, and L-nX contributed to the writing of the manuscript. All authors reviewed the manuscript.

## Funding 

This research was supported by the National Natural Science Foundation of China (31772566), the State Scholarship Fund of China Scholarship Council (201808340031), the Key Research Projects of Natural Science in Anhui Colleges and Universities (KJ2017A334), and the Agricultural Science and Technology Innovation Program of China (ASTIP-IAS13).

## Conflict of Interest

The authors declare that the research was conducted in the absence of any commercial or financial relationships that could be construed as a potential conflict of interest.

The reviewer ZH declared a past co-authorship with one of the authors YM to the handling editor.

## Abbreviations

ZGA, zygotic gene activation; DEL, differentially expressed lncRNA; GO, gene ontology; KEGG, Kyoto Encyclopedia of Genes and Genomes; WGCNA, Weighted gene co-expression network analysis; TOM, topological overlap matrix.

## Supplementary Material

Supplemental Figure 1 Goat lncRNAs identification pipeline. **(A)** Overview of goat lncRNAs identification pipeline. **(B)** Venn diagram presentation for prediction of coding potential using three software identifications, including CPC analysis, CNCI analysis, and pfam protein domain analysis.

Supplemental Figure 2 Hierarchical cluster tree of all DELs modules.**(A)** Hierarchical cluster tree of all DELs modules in the goat embryo. Modules correspond to the branch and are denoted by the color strips under the tree.

Supplemental Figure 3 Major lncRNA-mRNA subnetworks of high correlation modules in each preimplantation stage. **(A**–**G)** The red circle represents lncRNAs and the orange V shape represents co-expression mRNAs, and the size is expressed in degrees.

Supplemental Table S1 Summary information of transcriptome.

Supplemental Table S2 Result files for candidate lncRNAs by Cufflinks.

Supplemental Table S3 Primer pairs used to qRT-PCR amplification.

Supplemental Table S4 All identified mRNAs during the seven stages.

Supplemental Table S5 All identified lncRNAs during the seven stages.

Supplemental Table S6 Different expressed lncRNAs (DELs) of across the seven stages.

Supplemental Table S7 The GO enriched term of DELs (8- vs 16-cell stage).

Supplemental Table S8 The GO enriched term of DELs (oocytes vs 2-cell embryos).

Supplemental Table S9 Number of DELs (FPKM > 0.01 at least one sample during seven stages) contained in the stage high correlation modules.

Supplemental Table S10 GO enriched terms for the corresponding stages of the high correlation modules.

Supplemental Table S11 Target genes for each module of hub-lncRNAs.
