# Supplementary figures and images for: Identification of lncRNAs by RNA Sequencing Analysis During in Vivo Pre-Implantation Developmental Transformation in the Goat
Source: Front Genet. 2019 Oct 25;10:1040. doi: 10.3389/fgene.2019.01040 (PMC6823246; doi:10.3389/fgene.2019.01040)

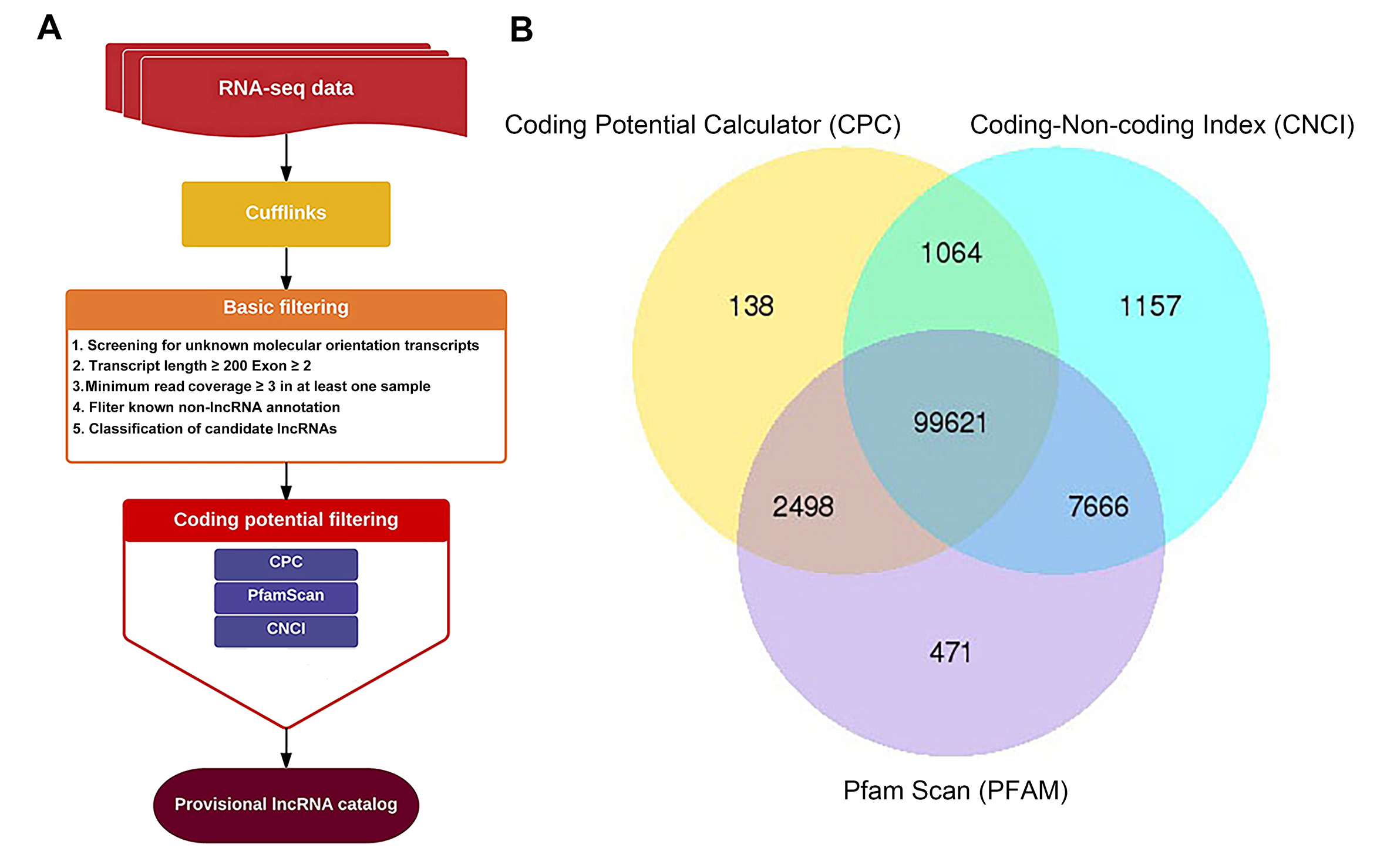

Supplement: Supplemental Figure 1 — Goat lncRNAs identification pipeline. (A) Overview of goat lncRNAs identification pipeline. (B) Venn diagram presentation for prediction of coding potential using three software identifications, including CPC analysis, CNCI analysis, and pfam protein domain analysis. [file Image_1.jpeg]

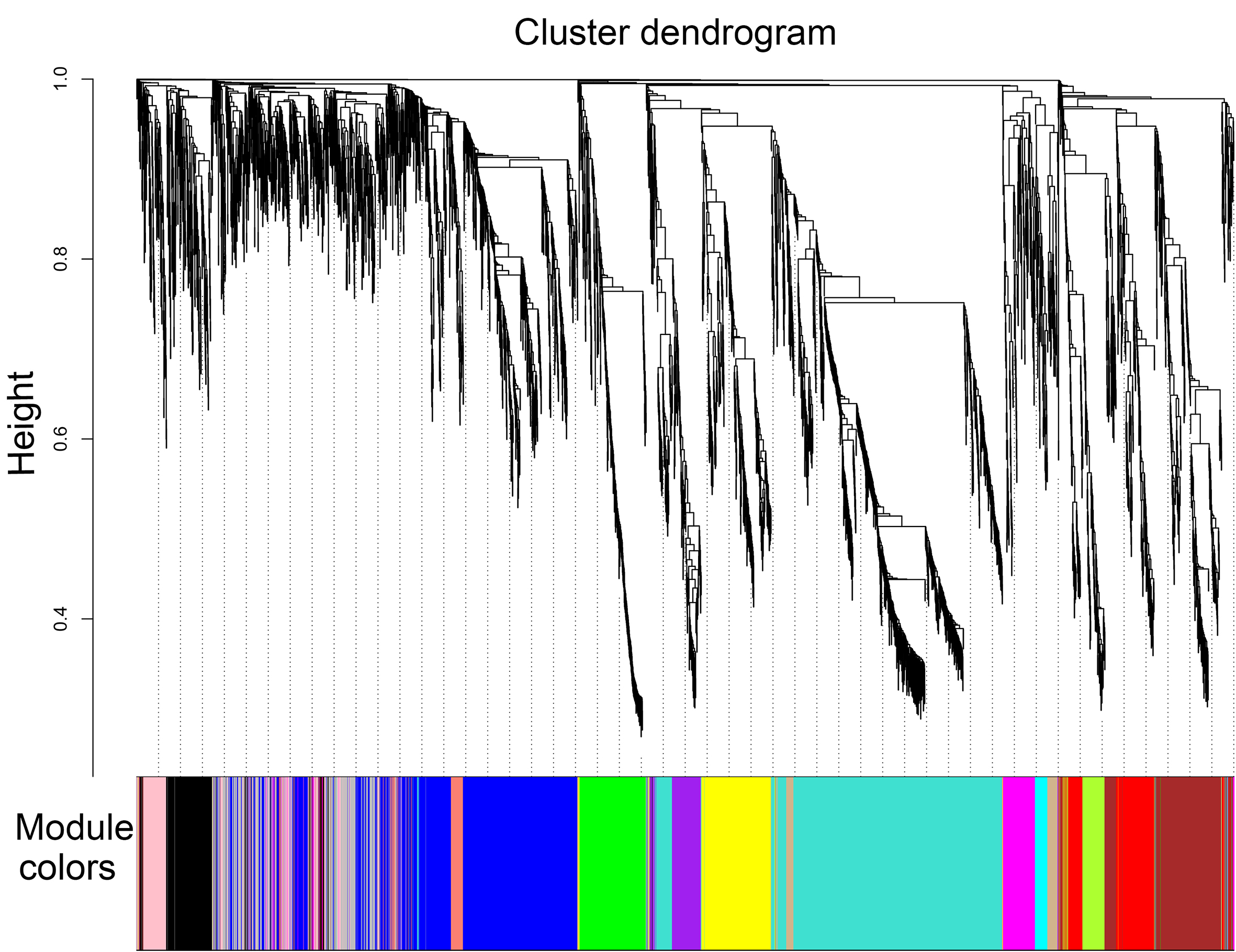

Supplement: Supplemental Figure 2 — Hierarchical cluster tree of all DELs modules.(A) Hierarchical cluster tree of all DELs modules in the goat embryo. Modules correspond to the branch and are denoted by the color strips under the tree. [file Image_2.jpeg]

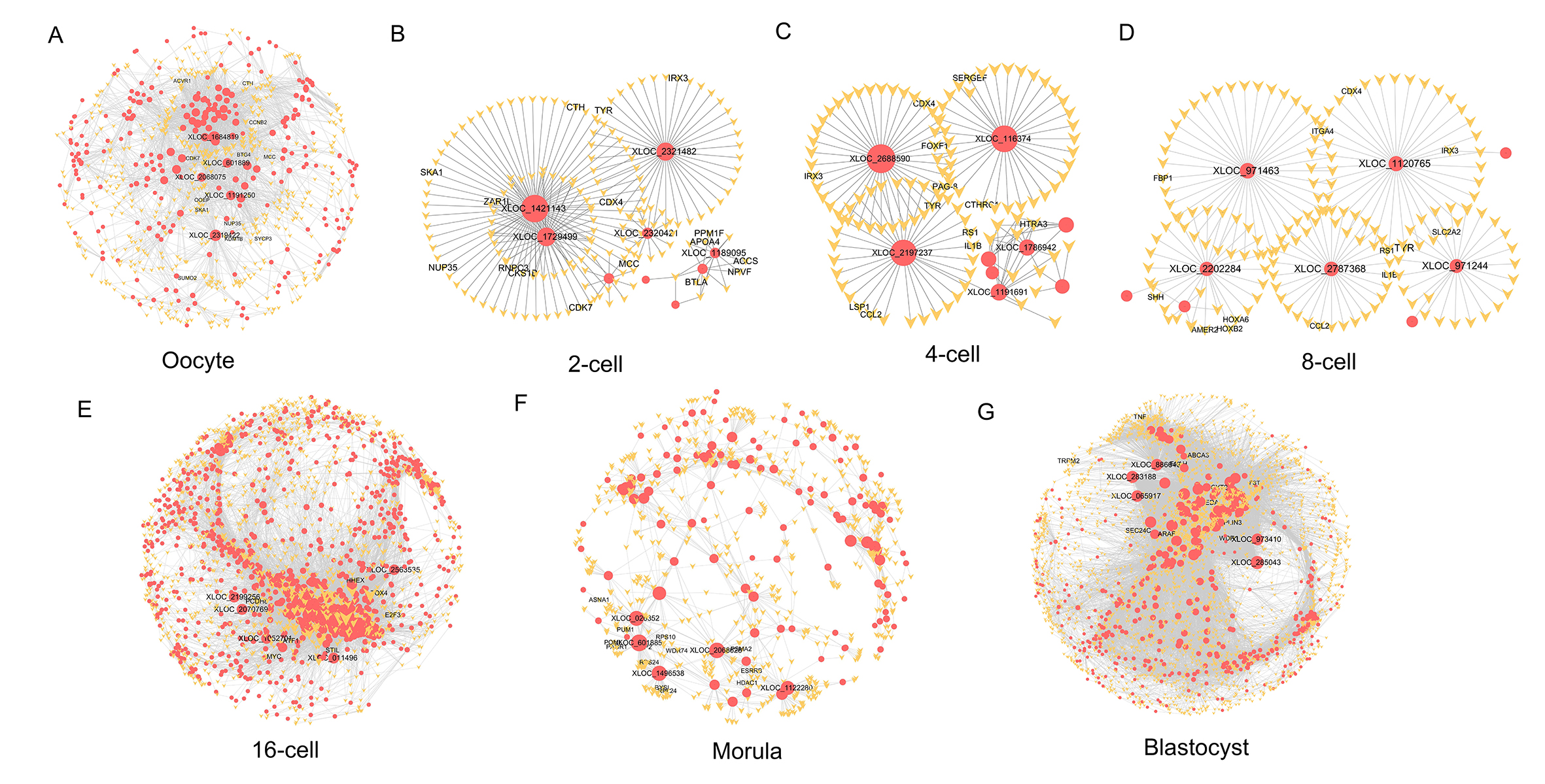

Supplement: Supplemental Figure 3 — Major lncRNA-mRNA subnetworks of high correlation modules in each preimplantation stage. (A–G) The red circle represents lncRNAs and the orange V shape represents co-expression mRNAs, and the size is expressed in degrees. [file Image_3.jpeg]
